# The relationship between rotator cuff integrity and acromiohumeral distance following open and arthroscopic rotator cuff repair

**DOI:** 10.1051/sicotj/2021012

**Published:** 2021-03-26

**Authors:** Erica Kholinne, Jae-Man Kwak, Yucheng Sun, Hyojune Kim, Dongjun Park, Kyoung Hwan Koh, In-Ho Jeon

**Affiliations:** 1 Faculty of Medicine, Trisakti University, Department of Orthopedic Surgery, St. Carolus Hospital 11440 Jakarta Indonesia; 2 Department of Orthopedic Surgery, Asan Medical Center, University of Ulsan 05505 Seoul Republic of Korea; 3 Department of Hand Surgery, Affiliated Hospital of Nantong University, Nantong, Nantong University 226001 Jiangsu PR China

**Keywords:** Propensity-matched, Regional acromion-humeral distance, Open repair, Arthroscopy

## Abstract

*Background*: Acromiohumeral distance (AHD) has become both a diagnostic and prognostic parameter related to rotator cuff pathology which is always measured in a 2-dimensional plane. The purposes of this study were (1) to evaluate the regional AHD with MRI following open and arthroscopic rotator cuff repair and, (2) to investigate its association to the rotator cuff integrity following medium to large size rotator cuff repair with open and arthroscopic manner. *Methods*: A retrospective review of 112 patients who were treated for full-thickness medium to large size rotator cuff tears either by open repair (open group) or arthroscopic repair (arthroscopic group) was done. All patients included in the study are those with at least 12 and 18 months for the post-operative MRI and clinical follow-up. Propensity score matching was used to select controls matched for age, sex, body mass index, tear size, and affected site. There were 56 patients in each group with a mean age of 63.3 years (range, 50 to 77 years). The post-operative functional and radiologic outcomes for both groups were compared. AHD was measured at three regions of interest (ROI) with MRI and compared pre-and post-operatively. *Results*: AHD was significantly greater in the open group when measured at the anterior third of the lateral acromion border compare to the arthroscopic group (*p* = 0.005). The re-tear rate was affected by AHD at the anterior third of the lateral border of the acromion for the arthroscopic and open group (*p* = 0.021, *p* = 0.029). The AHD measured at the anterior and middle third of lateral acromion border were significantly greater in healed compared to the re-tear rotator cuff group (*p* = 0.019, *p* = 0.022). *Conclusions*: Open rotator cuff repair showed greater AHD at the anterior third of the lateral border of the acromion. Regional AHD measured at anterior third of the lateral border of acromion significantly associated with rotator cuff integrity following repair. *Level of evidence*: propensity-matched case-control (Level II)

## Introduction

The Acromiohumeral distance (AHD) has been commonly used to measure the subacromial space which serves as both diagnostic and prognostic interest [1]. Loss of the normal AHD (< 6 mm) indicates full-thickness tear with high specificity [2] and is also a sign of poor prognosis following rotator cuff tear repair [3]. The measurement of AHD in conventional radiograph has been measured in the single 2-dimensional plan which needs a controlled X-ray beam of 20° caudally in anteroposterior projection. All radiographs should be taken under magnification control with the arm in neutral rotation. Although it was considered to be reproducible [4], still there is a chance of misinterpretation due to the need of having a specific setting to execute the position. A systematic review also mentioned that the reliability of AHD measurement using conventional radiographs has not been well supported due to conflicting results [5]. Instead, there is moderate evidence to support the reliability of AHD measurement using magnetic resonance imaging (MRI). The use of MRI for estimating the superior translation of the humeral head is currently under great discussion [6]. One of the issues is that the MRI is performed in dorsal decubitus position that will lead to overestimation of the measurement by gravity effect. Nevertheless, a previous study has confirmed that the assessment of AHD using MRI was not influenced by the force of gravity [6]. Hence, MRI may serve as an equal rotator cuff assessment tool to conventional radiograph with the benefit of assessing soft tissue integrity at once. Although MRI is considered a 3-dimensional imaging tool, nevertheless the measurement of AHD is done in a single 2-dimensional plane which is also similarly performed in the conventional radiograph.

Acromial morphologic was described as mostly curved type anteroposteriorly as seen in lateral scapular outlet projection [7]. The acromial morphologic is considered as one of the extrinsic factors in the development of rotator cuff pathology [8]. Therefore, the measurement of AHD as a diagnostic and prognostic radiographic parameter related to rotator cuff pathology should also consider the 3-dimensional acromial morphologic shape.

The primary aim of the current study was to investigate the regional AHD by using MRI before and after open and arthroscopic rotator cuff repair. The secondary aim of the current study was to investigate the relationship between regional AHD and rotator cuff integrity following open and arthroscopic rotator cuff repair. We hypothesized that (1) regional AHD would be greater following open rotator cuff repair, and (2) regional AHD has a significant association with rotator cuff integrity following open and arthroscopic rotator cuff repair.

## Methods

This retrospective study was designed as a matched case-control study with a propensity score matching technique.

### Patient selection

We included 1380 patients who underwent either open or arthroscopic rotator cuff repair between 2012 and 2016 in Asan Medical Center, Seoul, Korea. Inclusion criteria were (1) full-thickness rotator cuff tears, (2) primary repair, (3) with at least 12 months MRI follow-up, and (4) with at least 18 months of clinical follow-up. Exclusion criteria were as follows (1) incomplete medical data (*n* = 30), (2) previous surgery at affected shoulder, (3) with small (< 1 cm), and massive tears size (> 5 cm) were excluded from this study, (4) with concurrent subscapularis tear, acromioclavicular arthritis that requires concurrent distal clavicle resection, superior labral lesions that require concurrent repair, long head biceps pathology which need tenodesis, severe glenohumeral arthritis, anterior glenohumeral instability, (5) bilateral rotator cuff tear, (6) filled worker compensation.

### Surgical technique

The patients were positioned in the beach-chair position under general anesthesia, with the addition of interscalene block to reduce postoperative pain. Examination under anesthesia was performed prior to the surgical procedure to assess a passive range of motion. The surgical method was performed according to the two performing surgeons (each surgeon is only dedicated to one surgical method).

### Arthroscopic repair technique

A standard posterior portal was created 2 cm inferior and 1 cm medial to the posterolateral acromion corner. The anterior portal through rotator interval was introduced with the outside-in technique. A standard diagnostic round was performed. The arthroscope was then introduced to subacromial space to assess the acromion undersurface. An anterolateral acromioplasty was routinely performed in all patients. Afterward, a lateral portal was created under direct vision with the help of a spinal needle which later serves as the main viewing portal. A bursectomy was done to expose the rotator cuff tear and shape. The mobility of the rotator cuff was evaluated with a retriever. The edge of the rotator cuff was refreshed and trimmed with an arthroscopic shaver and or punch. The size of the tear was measured. The greater tuberosity was then prepared with burr with respect to remnant tissue. The number of anchors used was dependent on the size of the rotator cuff tear and the repair configuration (single or double row). The decision to use single or double row repair configuration was based on performing surgeon judgement. In the single-row repair configuration, the rotator cuff was routinely fixed with a bio-composite PEEK anchor (Helicoil PK^®^ 4.5 mm, Smith & Nephew, MA, USA). In the double-row repair configuration, the rotator cuff was routinely fixed with bio-composite PEEK anchor at medial row (Helicoil PK^®^ 4.5 mm, Smith & Nephew, MA, USA) and lateral row (Footprint Ultra PK^®^ 4.5 mm, Smith & Nephew, MA, USA). An attempt was always aimed to avoid overextension repair with the maximum surface coverage to the footprint at the greater tuberosity.

### Open repair technique

**Figure 1:**
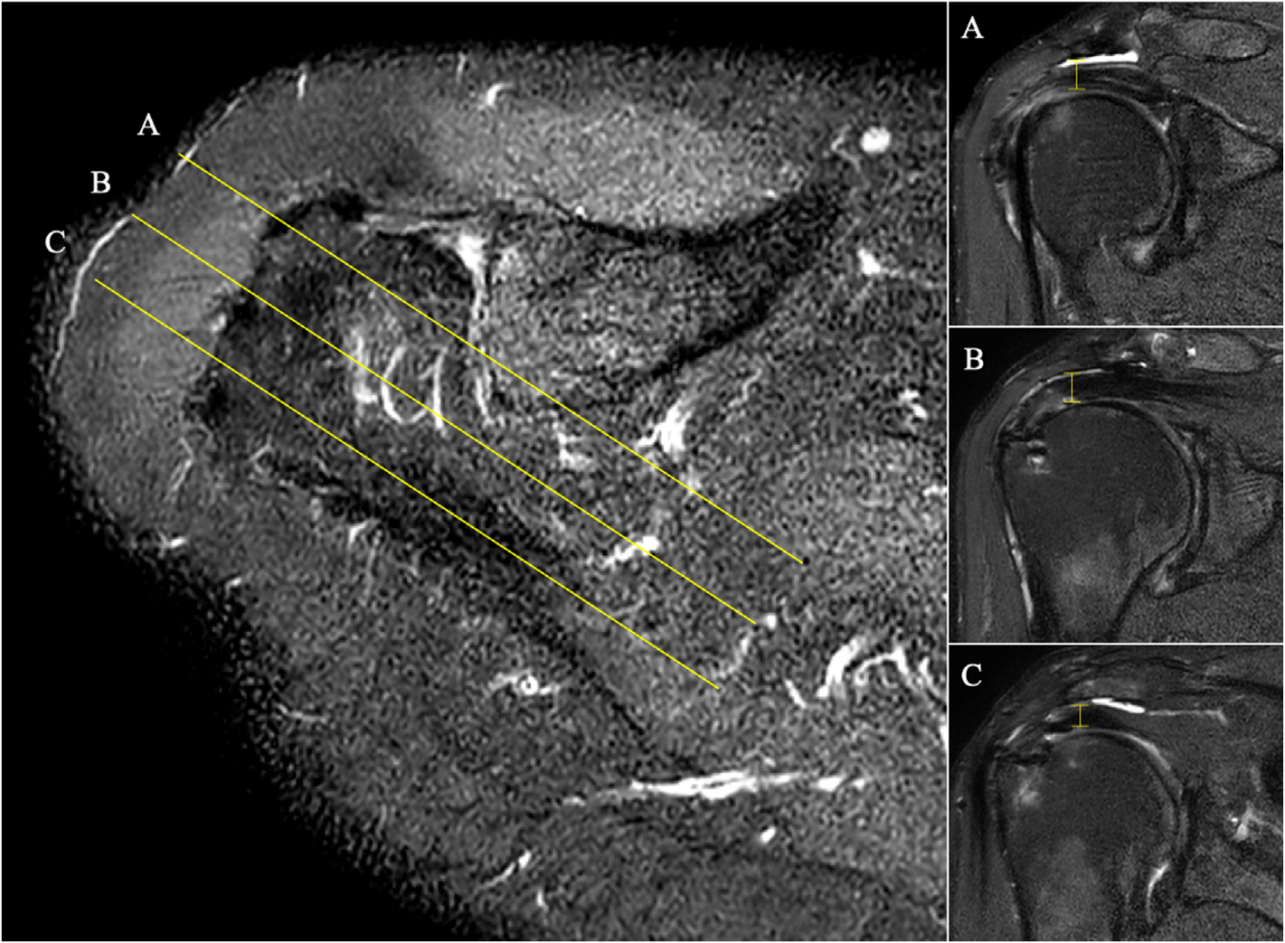
Axial view of the shoulder MRI showed regional AHD measurement which was performed in three ROI: anterior third (A), middle third (B), and posterior third (C) of lateral acromion border according to the scapular plane.

A 5-cm skin incision was longitudinally made starting from the mid-point of 1/3 lateral margin of the acromion to the lateral border of the coracoid process. The deltoid was split longitudinally about 3–4 cm between the anterior and middle deltoid. A curvilinear incision was made to take down a small portion of the anterior deltoid, and the coracoacromial ligament was peeled off from the undersurface of the acromial spur and preserved for reattachment later. An anterolateral acromioplasty was routinely performed with an oscillating saw. Multiple non-absorbable traction sutures no 2.0 Mersilk^®^ (Ethicon, Cincinnati, Ohio, USA) were placed on the edge of the torn rotator cuff to assist the mobilization of the tendon. Gentle release of adhesion and removal of bursal hypertrophy was carried out using Mayo scissors with respect to the remnant rotator cuffs. Once adequately mobilized, the margin was converged with multiple tendon-to-tendon sutures when necessary and the torn edge of the tendon was reattached to the greater tuberosity by No. 2 Ethibond^®^ (Ethicon, Cincinnati, Ohio, USA) in trans-osseous double mattress fashion (Figure 1). Deltoid muscle insertion and coracoacromial ligament were repaired to the acromion with heavy absorbable suture no. 1 Vicryl^®^ (Ethicon, Cincinnati, Ohio, USA).

### Postoperative protocol

All patients’ arms remained in a sling for 6 weeks, and they were allowed only passive range of motion during this time period. At 6 weeks, a gradual full active motion was started, progressing to resistive strengthening, which was continued for a total of 3–4 months. Heavy labor activities were restricted until 6 months.

### Clinical outcome assessment

An independent nurse practitioner documented the clinical assessment of pre-operative and post-operative parameters for (1) pain score with visual analog score (VAS), (2) functional outcome with age-adjusted Constant score and American Shoulder and Elbow Surgeons (ASES) score, (3) Range of motion (forward elevation and external rotation) with a hand-held goniometer and (4) muscle power (Abduction, supraspinatus and external rotator muscle strength) with myometer (Mecmesin Co., Nottingham, UK). The complication following surgery was also recorded.

### Radiological outcome assessment

All patients underwent radiological assessment with a 3.0-T MRI at a minimum of 1 year following rotator cuff repair. Rotator cuff integrity was evaluated according to Sugaya et al. [9]. The fatty infiltration for supraspinatus and infraspinatus were evaluated according to Fusch et al. [10]. Plain radiographs were used to determine acromial index [11] and critical shoulder angle [12] as described by previous studies. AHD was evaluated with MRI according to Werner et al. [13]. AHD was measured in three zones (anterior third, middle third, and posterior third) at the lateral acromion border according to the scapular plane (Figure 1). The evaluation was done independently by two shoulder fellowship-trained orthopedic surgeons (EK and JMK) and any discrepancies were resolved in a consensus meeting; if a disagreement persisted, a senior shoulder surgeon which was not involved in the surgery (KHK) were consulted for the final assessment. All radiologic parameters were recorded pre-and post-operatively.

### Statistical analysis

Sample size calculation with the power of 90% and 0.05 two-sided significance level was performed with the minimum expected clinical important difference in means of constant shoulder score for 10.4 points [14]. The propensity score matching technique was done with age, gender, affected shoulder, BMI as covariates. A total of 1380 cases were included. We excluded 30 patients because of incomplete medical data. Of the included 1350 patients, 543 had open rotator cuff repair (837 had arthroscopic rotator cuff repair). Of the 543 patients with open rotator cuff repair, only 91 patients were with at least 18 months of clinical follow-up. Of the 91 patients, 56 patients were with at least 1-year MRI follow-up. The controls are arthroscopic groups matched for age, sex, BMI, tear site, and the affected site selected by propensity score matching.

Test for normality using Kolmogorov-Smirnov was applied to all data set prior to statistical analysis. Mann–Whitney U test was used to compare data set with skewed distribution, meanwhile, an independent *t*-test was used to compare data set with a normal distribution. The significance level was set at *p* < 0.05. The statistical analysis was performed with Statistical Package for the Social Sciences software (v. 23.0; SPSS, Inc., Chicago, IL, USA). Statistical analysis was conducted with the supervision of a biostatistician.

## Results

### Patients demographics and preoperative baseline data

A total of 112 patients with 24.2 months (range, 19–26 months) follow-up were included for analysis. The open group and arthroscopic group patients’ characteristics and preoperative baseline data are described in Table 1. There was no significant difference in demographic characteristics between the two matched groups.

**Table 1:** Patients demographics and preoperative baseline data for both group.

Variable	Open group	Arthroscopic group	*P*-value
	(*n* = 56)	(*n* = 56)	
Age (mean ± SE)	63.6 ± 7.9	61.5 ± 5.5	0.111
Gender	F = 37 (66.7%)	F = 33 (59.3%)	0.109
	M = 19 (33.3%)	M = 23 (40.7%)	
Affected shoulder			
Right	43 (77.8%)	48 (85.2%)	0.060
Left	13 (22.2%)	8 (14.8%)	
Body mass index			
Underweight (<18.5)	0	0	0.805
Normal (18.5 – 24.9)	29 (51%)	25 (44.6%)	
Overweight (25.0 – 29.9)	27 (49%)	28 (50%)	
Obese class I (30.0 – 34.9)	0	3 (5.4%)	
Obese class II (35.0 – 39.9)	0	0	
Obese class III (>= 40.0)	0	0	
Range of motion (ROM) (mean ± SE)			
Forward Elevation (FE)	139.6 ± 5.1	144.2 ± 3.6	0.468
External Rotation (ER)	43.5 ± 4.1	43.1 ± 7.7	0.827
Functional score			
Constant score (mean ± SE)	54.1 ± 3.6	56.1 ± 2.7	0.657
ASES score (mean ± SE)	57.1 ± 3.9	58.6 ± 3.3	0.774
Muscle power (mean ± SE)			
Abd	3.6 ± 0.3	2.8 ± 0.3	0.156
SST	2.9 ± 0.3	2.9 ± 0.3	0.913
ER	4.0 ± 0.3	3.1 ± 0.2	0.58
Pain (VAS) (mean ± SE)	4.8 ± 0.3	5.5 ± 0.3	0.14
Tear size			
Medium	43 (76.7%)	43 (76.7%)	1.000
Large	13 (23.3%)	13 (23.3%)	
Fatty infiltration (mean ± SE)			
Supraspinatus	0.4 ± 0.6	0.7 ± 0.7	0.192
Infraspinatus	0.5 ± 0.7	0.7 ± 0.5	0.283
Acromiohumeral distance (mm) (mean ± SE)			
At anterior third of lateral acromion border	4.8 ± 1.4	5.2 ± 0.9	0.640
At middle third of lateral acromion border	4.7 ± 1.2	4.8 ± 0.7	0.676
At posterior third of lateral acromion border	5.9 ± 1.2	5.8 ± 0.8	0.957
Acromial Index	0.8 ± 0.1	0.8 ± 0.1	0.687
Critical Shoulder Angle	37.8 ± 3.8	36.8 ± 3.7	0.141

VAS = Visual Analog Scale; ASES = American Shoulder and Elbow Surgeons score; ROM = range of motion; FE = forward elevation; ER = external rotation; SST = supraspinatus; ER = external rotator; Abd = abduction.

### Clinical and radiological outcome assessment

The clinical outcome consisted of ROM, Constant score, ASES score, muscle power, and VAS were significantly improved following surgery at the final follow up. Table 2 described the comparison of clinical and radiological outcomes between the open and arthroscopic groups. The arthroscopic group showed significantly better ROM (*p* = 0.006) and VAS score (*p* < 0.001) compared to the open group. On the other hand, the open group showed significantly better constant and ASES score (*p* = 0.012 and *p* = 0.047). The muscle power for abduction, supraspinatus and external rotation were more superior in open group with no statistical difference (*p* = 0.068, *p* = 0.626, and *p* = 0.182). No complications were seen in both groups.

**Table 2:** Postoperative clinical and radiographic data of both group.

Variable	Open group	Arthroscopic group	*P*-value
Range of motion (ROM)			
Forward Elevation (FE)	151.8 ± 0.9	158.3 ± 2.0	0.006*
External Rotation (ER)	44.2 ± 0.3	50.9 ± 2.1	
Functional score			
Constant score	75.9 ± 1.8	69.5 ± 1.5	0.012*
ASES score	89.1 ± 1.3	85.3 ± 1.3	0.047*
Muscle power (kg)			
Abd	4.4 ± 0.4	3.4 ± 1.5	0.068
SST	3.9 ± 0.4	3.7 ± 0.2	0.626
ER	4.4 ± 0.3	3.9 ± 0.1	0.182
Pain (VAS)	1.7 ± 0.2	0.5 ± 0.1	< 0.001*
Fatty infiltration			
Supraspinatus	0.3 ± 0.5	0.5 ± 0.6	0.181
Infraspinatus	0.5 ± 0.7	0.6 ± 0.5	0.182
Acromiohumeral distance (mm)			
At anterior third of lateral acromion border	7.9 ± 1.6	7.1 ± 1.2	0.005*
At middle third of lateral acromion border	7.2 ± 1.8	6.7 ± 1.1	0.133
At posterior third of lateral acromion border	6.6 ± 1.3	6.4 ± 1.4	0.377
Postoperative rotator cuff integrity			
Sugaya type I	28 (50%)	26 (46.4%)	
Sugaya type II	18 (32.1%)	18 (32.1%)	0.642
Sugaya type III	8 (14.3%)	8 (14.2%)	
Sugaya type IV	None	4 (7.3%)	
Sugaya type V	2 (3.6%)	None	
Acromial Index	0.69 ± 0.06	0.76 ± 0.22	0.020*
Critical Shoulder Angle	34.8 ± 3.6	33.6 ± 4.3	0.128

All value described as mean and standard error. VAS = Visual Analog Scale; ASES = American Shoulder and Elbow Surgeons score; ROM = range of motion; FE = forward elevation; ER = external rotation; SST = supraspinatus; ER = external rotator; Abd = abduction.

* Significant level = *P* < 0.05.

The fatty infiltration of supraspinatus and infraspinatus showed no significant difference between both groups. The mean AHD following open rotator cuff repair was measured as 7.9 ± 1.6 mm (at anterior third), 7.2 ± 1.8 mm (at middle third), and 6.6 ± 1.3 mm (at posterior third) of the lateral border of the acromion. The mean AHD following arthroscopic rotator cuff repair was measured as 7.1 ± 1.2 mm (at anterior third), 6.7 ± 1.1 mm (at middle third), and 6.4 ± 1.4 mm (at posterior third) of the lateral border of the acromion. AHD was significantly greater in the open group when measured at the anterior third of the lateral acromion border compare to the arthroscopic group (*p* = 0.005). The re-tear rate was higher in the arthroscopic group (21.5%) compare to the open group (17.9%) with no statistical difference (*p* = 0.300).

**Figure 2:**
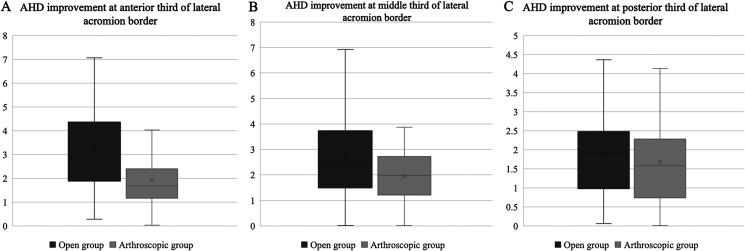
Boxplot of AHD improvement measured at anterior third (A), middle third (B), and posterior third (C) of lateral acromion border.

The AHD is significantly improved in all zone of measurement following both open and arthroscopic rotator cuff repair as shown in Table 3. The improvement of AHD was observed to be greater in an open group with an average of 78.4% at the anterior third, 60.9% at the middle third, and 44.5% at the posterior third of the lateral acromion border. The improvement of AHD was relatively lower in the arthroscopic group with an average of 42.5% at the anterior third, 39.8% at the middle third, and 32.4% at the posterior third of the lateral acromion border. Figure 2 summarized the AHD improvement of all regions of interest (ROI) measured. The improvement of AHD was higher at the anterior, middle, and posterior third of the lateral acromion border (*p* < 0.001, *p* < 0.001, *p* = 0.09) in the open group compare to the arthroscopic group.

**Table 3:** The improvement of AHD for both group.

AHD (mm)	Preoperative	Post-operative	Average AHD improvement	*P*-value
	(mean ± SD)	(mean ± SD)	(mean ± SD) (%)	
Open Group				
At anterior third of lateral acromion border	4.8 ± 1.4	7.9 ± 1.6	3.1 ± 1.8 (78.4%)	<0.001*
At middle third of lateral acromion border	4.7 ± 1.2	7.2 ± 1.8	2.4 ± 1.8 (−60.90%)	<0.001*
At posterior third of lateral acromion border	5.9 ± 1.2	6.6 ± 1.3	1.5 ± 1.2 (−44.50%)	<0.001*
Arthroscopic Group				
At anterior third of lateral acromion border	5.2 ± 0.9	7.1 ± 1.2	2.1 ± 1.4 (−42.50%)	<0.001*
At middle third of lateral acromion border	4.8 ± 0.7	6.7 ± 1.1	1.9 ± 1.1 (−39.80%)	<0.001*
At posterior third of lateral acromion border	5.8 ± 0.8	6.4 ± 1.4	1.5 ± 1.4 (−32.40%)	<0.001*

* Paired *t*-test.

A subgroup analysis of clinical and radiological parameters was performed in regards to the repair integrity as shown in Table 4. Clinical outcomes for VAS score, constant score, ASES score, and active forward flexion were significantly superior in the healed rotator cuff group. The AHD measured at the anterior and middle third of lateral acromion border were significantly greater in healed compared to the re-tear rotator cuff group (*p* = 0.019, *p* = 0.022). The AHD measured at the posterior third of the lateral acromion border is comparable without a significant *P*-value (*p* = 0.994).

**Table 4:** Subgroup analysis of clinical and radiological parameters for healed versus re-tear rotator cuff.

Postoperative Parameters			Healed rotator cuff (*n* = 90)	Re-tear rotator cuff (*n* = 22)	*P*-value
Clinical	VAS score		1.9 ± 0.8	3.3 ± 0.6	0.012*
	Constant score		74.8 ± 4.5	49.3 ± 7.5	0.002*
	ASES score		79.8 ± 9.7	59.2 ± 11.3	<0.001*
	Active ROM	Forward flexion	151.3 ± 12.1	104.5 ± 17.4	<0.001*
		External rotation	36.2 ± 12.4	33.9 ± 11.1	0.082
Radiological	AHD (mm)	At anterior third of lateral acromion border	7.7 ± 2.1	4.9 ± 2.2	0.019*
		At middle third of lateral acromion border	7.5 ± 1.9	5.1 ± 2.1	0.022*
		At posterior third of lateral acromion border	7.0 ± 1.3	6.9 ± 1.4	0.994

* Independent *t*-test.

## Discussions

The AHD has been widely used as a parameter to represent the rotator cuff integrity [1]. The AHD has been measured commonly by the conventional radiograph [15–17] despite the likelihood of misinterpretation due to the need for a specific position. For this reason, the use of MRI has been described to overcome the limitation of the conventional radiographs. Despite the acromial shape, assessment of AHD with MRI has only been performed as a 2-dimensional plane which is considered equal to the conventional radiograph. Therefore, there is a need to assess the regional AHD as the representation of the 3-dimensional acromial morphologic shape in relation to the rotator cuff repair.

The current study found that open rotator cuff repair resulted in better functional outcomes with Constant and ASES scores as measured variables. The minimal clinically important difference is 10.4 for Constant score and a range of 12–17 for ASES score. Despite the significant statistical analysis that showed superiority in functional outcome following open rotator cuff repair, we think that differences between both groups did not exceed the smallest amount to be meaningful therefore this can be disregard and account for as a comparable outcome [18]. When we analyzed the correlation between clinical outcome score and the AHD improvement, no significant difference was found. We attributed to the reason that multiple factors play a role in the healing of rotator cuff as well as rotator cuff re-tears. The AHD is merely one extrinsic factor to be considered in rotator cuff pathology and healing.

The most significant limitation of this study is its retrospective nature. The included population is ideal for a clinical study, nevertheless, it may limit the extrapolation of findings to the general population, leading to the selective bias in this study. This study also only included medium to large size tear thus limit the translation to all rotator cuff tear size. Despite the limitations, we would like to minimize bias, therefore, excluding the small and massive rotator cuff tear from the study design to provide a straightforward result. We acknowledged that it is difficult to blind the radiologic evaluation process due to the drilled holes in the acromion following open rotator cuff repair as one of the study limitations. Lastly, the postoperative AHD measurement was not available in the early postoperative period in which may provide information to understand the correlation between retear and eventual variations of subacromial space.

Previous studies have been done to compare the clinical outcomes and tendon healing following arthroscopic or open rotator cuff repair. Most of them were conducted with the non-matched samples, enrolled in all sizes of rotator cuff tear (small to massive) with non-uniform repair technique for an open procedure [19–21]. The technique used in the open repair which is included in the previous studies is large in variability [20, 22, 23]. Postoperative integrity of rotator cuff for the previous studies has included a bedside ultrasonogram evaluation [24] or an open MRI scan [22]. Ultrasonogram is highly operator dependent while the accuracy of open MRI scan is inferior to the conventional MRI. The integrity of rotator cuff following 1-year postoperative was found to be superior in open repair technique compared to arthroscopic repair technique with no statistically significant difference. This finding is similar to those from Bayle et al. with ultrasonogram evaluation which reported a 7% to retear rate with arthroscopic repair versus a 9% retear rate following open repair despite the inclusion of small and massive size tear [24]. Bishop et al. showed that large size tear has better cuff integrity when repaired with open repair with 62% intact rate versus 24% with arthroscopic repair when evaluated with MRI [22]. The current study reported that the re-tear rates were 17.9% and 21.5% for arthroscopic and open rotator cuff repair for medium to large rotator cuff tear. The integrity of the rotator cuff in our center was evaluated using MRI considering the unpredictability of sonogram examination [25, 26] which perhaps reflected by the comparable retear rate reported by Bishop et al. For this reason, we thought that the MRI evaluation may serve as a reliable measurement tool to evaluate postoperative integrity.

Despite the limitations mentioned, the current study has several strengths. First, we include only medium to large size rotator cuff tear with an appropriate power analysis prior to the study. Second, MRI 3-Tesla was used to evaluate postoperative rotator cuff integrity. Third, this study ensures matching case and control group with the use of propensity score matching to balance the clinical characteristic of both groups, allowing more accurate comparisons within observational studies by simulating a randomized controlled trial [27]. The matching technique, which includes age, gender, affected site, BMI ensures the assignment of control to each case. This is the major advantage of the frequency matching technique will select nearest neighbors for control to each case despite the slight difference in matching variable distribution. Because of potential residual confounding, regression models were also controlled for age, sex, and body mass index [28, 29].

## Conclusions

Open rotator cuff repair showed greater AHD at the anterior third of the lateral border of the acromion. Regional AHD measured at anterior third of the lateral border of acromion significantly associated with re-tear rate following both open and arthroscopic rotator cuff repair. Nevertheless, regional AHD measured at the middle third of the lateral border of acromion is significantly associated only with re-tear rate following open rotator cuff repair.

## Conflict of interest

All authors had nothing to declare.

## Funding statement

The authors declare that no funding was involved in this study.
